# High prevalence of biochemical disturbances of chronic kidney disease - mineral and bone disorders (CKD-MBD) in a nation-wide peritoneal dialysis cohort: are guideline goals too hard to achieve?

**DOI:** 10.1590/2175-8239-JBN-2020-0147

**Published:** 2021-02-03

**Authors:** Rafael Weissheimer, Sergio Gardano Elias Bucharles, Cesar Augusto Madid Truyts, Vanda Jorgetti, Ana Elizabeth Figueiredo, Pasqual Barrett, Marcia Olandoski, Roberto Pecoits-Filho, Thyago Proença de Moraes

**Affiliations:** 1 Pontifícia Universidade Católica do Paraná, Escola de Medicina, Curitiba, PR, Brasil.; 2 Pontifícia Universidade Católica do Rio Grande do Sul, Programa de Pós-Graduação em Medicina e Ciências da Saúde, Porto Alegre, RS, Brasil.; 3 Universidade Estadual de São Paulo, Escola de Medicina, Botucatu, SP, Brasil.; 4 Universidade de São Paulo, Escola de Medicina, São Paulo, SP, Brasil.; 5 Universidade Federal do Paraná, Curitiba, PR, Brasil.

**Keywords:** Phosphorus, Calcium, Renal Insufficiency, Chronic, Fósforo, Cálcio, Insuficiência Renal Crônica

## Abstract

**Introduction:**

Chronic kidney disease - mineral and bone disorders (CKD-MBD) are common in dialysis patients. Definition of targets for calcium (Ca), phosphorus (P), parathormone (iPTH), and alkaline phosphatase (ALP) and their treatment recommendations, are provided by international guidelines. There are few studies analyzing CKD-MBD in peritoneal dialysis (PD) patients and the impact of guidelines on mineral metabolism control. The aim of our study was to describe the prevalence of biomarkers for CKD-MBD in a large cohort of PD patients in Brazil.

**Methods:**

Data from the nation-wide prospective observational cohort BRAZPD II was used. Incident patients were followed between December 2004 and January 2011. According to KDOQI recommendations, reference ranges for total Ca were 8.4 to 9.5 mg/dL, for P, 3.5 to 5.5 mg/dL, for iPTH, 150-300 pg/mL, and for ALP, 120 U/L.

**Results:**

Mean age was 59.8 ± 16 years, 48% were male, and 43% had diabetes. In the beginning, Ca was 8.9 ± 0.9 mg/dL, and 48.3% were on the KODQI target. After 1 year, Ca increased to 9.1 ± 0.9 mg/dL and 50.4% were in the KDOQI preferred range. P at baseline was 5.2 ± 1.6 mg/dL, with 52.8% on target, declining to 4.9 ± 1.5 mg/dL after one year, when 54.7% were on target. Median iPTH at baseline was 238 (P25% 110 - P75% 426 pg/mL) and it remained stable throughout the first year; patients within target ranged from 26 to 28.5%. At the end of the study, 80% was in 3.5 meq/L Ca dialysate concentration, 66.9% of patients was taking any phosphate binder, and 25% was taking activated vitamin D.

**Conclusions:**

We observed a significant prevalence of biochemical disorders related to CKD-MBD in this dialysis population.

## Resumo

**Introdução:**

Os distúrbios minerais e ósseos da doença renal crônica (DMO-DRC) são comuns em pacientes em diálise. A definição de metas para cálcio (Ca), fósforo (P), paratormônio (PTHi) e fosfatase alcalina (FA) e suas recomendações de tratamento são fornecidas por diretrizes internacionais. Há poucos estudos analisando o DMO-DRC em pacientes em diálise peritoneal (DP) e o impacto das diretrizes no controle do metabolismo mineral. O objetivo do nosso estudo foi descrever a prevalência de alterações nos marcadores para DMO-DRC em uma grande coorte de pacientes em DP no Brasil.

**Métodos:**

Foram utilizados dados da coorte observacional prospectiva nacional BRAZPD II. Pacientes incidentes foram acompanhados entre Dezembro de 2004 e Janeiro de 2011. De acordo com as recomendações do KDOQI, os intervalos de referência para Ca total foram de 8,4 a 9,5 mg/dL, para P, 3,5 a 5,5 mg/dL, para PTHi, 150-300 pg/mL, e para FA, 120 U/L.

**Resultados:**

A idade média foi de 59,8 ± 16 anos, 48% eram homens e 43% tinham diabetes. No início, o Ca era de 8,9 ± 0,9 mg/dL, e 48,3% estavam na meta do KODQI. Após 1 ano, o Ca aumentou para 9,1 ± 0,9 mg/dL e 50,4% estavam na faixa preferida do KDOQI. P basal era 5,2 ± 1,6 mg/dL, com 52,8% na meta, diminuindo para 4,9 ± 1,5 mg/dL após um ano, quando 54,7% estavam na meta. O PTHi basal mediano foi de 238 (P25% 110 - P75% 426 pg/mL) e permaneceu estável durante o primeiro ano; os pacientes dentro da meta variaram de 26 a 28,5%. No final do estudo, 80% estavam na concentração de 3,5 meq/L de Ca dialisato, 66,9% dos pacientes estavam tomando qualquer quelante de fosfato, e 25% estavam tomando vitamina D ativada.

**Conclusões:**

Observamos uma prevalência significativa de distúrbios bioquímicos relacionados ao DMO-DRC nesta população em diálise.

## Introduction

Chronic kidney disease-mineral and bone disorders (CKD-MBD) are considered some of the most common complications in dialysis patients, with important impact on patient morbidity and mortality^1^^-^ 3. Management of CKD-MBD, particularly (especially) the definition of targets for biochemical parameters, namely calcium, phosphorus, parathormone, alkaline phosphatase and their treatment recommendations, are supported by current guidelines^4^^,^ 5.

The majority of studies focusing on CKD-MDB in dialysis patients have involved patients on hemodialysis. However, studies with patients on chronic peritoneal dialysis (PD) showed strong evidence that abnormalities of mineral metabolism are also associated with all-cause, cardiovascular^6^, and infection-related mortality^7^. Another large national population-based longitudinal study found that in PD Chinese patients population, both hyper and hypophosphatemia and elevated alkaline phosphatase were associated with increase mortality^8^.

Full compliance to every recommendation for CKD-MBD among dialysis patients is not always feasible. For example, when two of the most prescribed drugs to control mineral and bone disorders are used (calcitriol and calcium-based phosphate binders) aiming at reduction of iPTH and phosphate control, a single patient may experience hypercalcemia and/or hyperphosphatemia and move out from guidelines’ recommended range.

The National Kidney Foundation Kidney Diseases Outcomes Quality Initiative (NKF-KDOQI) guideline for bone metabolism in CKD recommends that serum levels of phosphorus of dialysis patients should be maintained between 3.5 and 5.5 mg/dL. For total serum calcium levels, the recommendation is to keep the value preferentially between 8.4 to 9.5 mg/dL^9^^-^ 11. Similarly, the KDIGO (Kidney Disease Improving Global Outcomes) guidelines suggest lowering elevated phosphorus levels toward the normal range and avoiding hypercalcemia^5^.

The background for such recommendations is therefore clear, being both calcium and phosphorus abnormalities in CKD patients strongly associated with vascular calcification and cardiovascular and overall mortality^1^^,^ 12. Interestingly, the literature lacks information about whether the publication of these guidelines was effective to reduce the prevalence of hyper and hypophosphatemia, and hyper and hypocalcemia in dialysis population, and the impact of those guidelines in peritoneal dialysis patients. Indeed, adherence to all the recommended targets and the application of appropriate pharmacological strategies may result in biochemical abnormalities, as can occur in patients that receive calcitriol to treat secondary hyperparathyroidism but develop hypercalcemia and/or hyperphosphatemia.

For intact parathormone (iPTH), KDIGO guidelines suggest maintaining levels between 2 to 9 times the upper limit, and KDOQI guidelines suggest levels between 150 to 300 pg/mL. Regarding alkaline phosphatase (ALP), there are no suggested values, only the information that altered levels are related to remodeling disturbances and that levels should be monitored^5^^,^ 10.

The aim of our study was to describe the prevalence of traditional biochemical parameters of bone-mineral disorders in PD patients, based on the values proposed by the KDOQI guideline, along the first year of therapy, in a large cohort of advanced CKD patients in Brazil.

## Methods

This is a nation-wide prospective observational cohort study that used data from the Brazilian Peritoneal Dialysis Study II (BRAZPD II). Socio-demographic, clinical, and laboratory characteristics of the population were previously published^13^. The ethical committees of all participating centers approved the study. In summary, our database contains clinical and laboratory information from 122 dialysis centers of all five geographic regions of Brazil, corresponding to 65 to 70% of all prevalent PD patients in the country during the study period. Patients were included in this study and followed-up between December 2004 and January 2011.

In addition to the general demographic and clinical characteristics we also reported the Davies score for the population. This is a traditional score used on PD studies and is simple to calculate. The score considers the presence of up to 11 comorbidities, each one accounting for 1 point. These comorbidities are malignancy, ischemic heart disease, peripheral vascular disease, left ventricular dysfunction, diabetes, systemic collagen vascular disease, chronic obstructive lung disease, pulmonary fibrosis, active pulmonary tuberculosis, asthma, and cirrhosis^14^^,^ 15.

The main goal of our study was to describe the prevalence of patients meeting the CKD-MBD KDOQI preferential range of biochemical variables, because this guideline was current at that time, especially for calcium and phosphorus targets, for patients after one year of initiation of chronic PD. For this study, we included all incident patients (those who started PD during the study) that remained at least 90 days in therapy. Calcium and phosphorus levels were measured and recorded monthly following local regulatory rules and proper laboratory methodologies. Patients were stratified in groups according to serum levels of calcium, phosphorus, and iPTH according to KDOQI recommendation: the reference value for total calcium was 8.4 to 9.5 mg/dL, and for phosphorus, 3.5 to 5.5 mg/dL. We also explored the results of iPTH and alkaline phosphatase, although the frequency of measurement of iPTH was only every 6 months. For iPTH, we considered the target proposed by the guideline available at the time (150-300 pg/mL) and for ALP, the value of 120 U/L, which is reported in other references. Information on patient’s prescriptions was also collected. All the biochemical variables related to mineral and bone disorders were obtained at baseline, 6 months, and 12 months after PD initiation.

### Statistical analysis

Continuous variables are reported as mean ± SD or median and range, while categorical variables (e.g., gender, race, etc.) are reported as frequencies or percentages. The comparison between continuous variables was performed using the paired T-test and for categorical values, the chi-square test. It is important to mention beforehand that given the large sample size of the BRAZPD II, differences between variables normally reach statistical significance and clinical relevance should be discussed with this view in mind. Analysis was performed using STATA 14 and figures were generated in the Excel program.

## Results

### Baseline characteristics

The mean age of the study population was 59.8 ± 16 years, 48% were male, 71% had history of hypertension, and diabetes was present in 43% of the patients. Thirty-seven percent had history of previous hemodialysis, 49% received pre-dialysis care, and 64% were caucasians. Baseline characteristics of the study population, including comorbidity Davies score, which was previously described (14), are presented in Table 1.

**Table 1:** Clinical and demographic characteristics of patients

Variable	Incident patients (n = 7,007)
Age (years)	59.8 ± 16.2
Male	48%
Diabetes mellitus	43%
Previous hemodialysis	37%
Arterial hypertension	71%
Pre-dialysis care	49%
BMI	
< 18.5	7%
18.5 - 24.9	51%
≥ 25	42%
Davies Score	
0	37%
1 - 2	57%
3 - 4	6%
Family income	
< 2 MW	34%
2 - 5 MW	46%
> 5 MW	20%
Race	
White	64%
Black	12%
Others	24%
Distance from dialysis center	
< 25 km	58%
25 - 100 km	32%
> 100 km	10%
Primary renal disease	
Diabetes	36%
Hypertension	16%
Chronic glomerulonephritis	9%
Unknown	22%
Others	17%
Education level	
Up to 4 years	66%
More than 4 years	34%
Center experience (patient-year)	
≤ 11	8%
11,1 - 25	25%
> 25	65%

BMI: body mass index; MW: minimal wage in Brazil.

### Calcium

**Figure 1:**
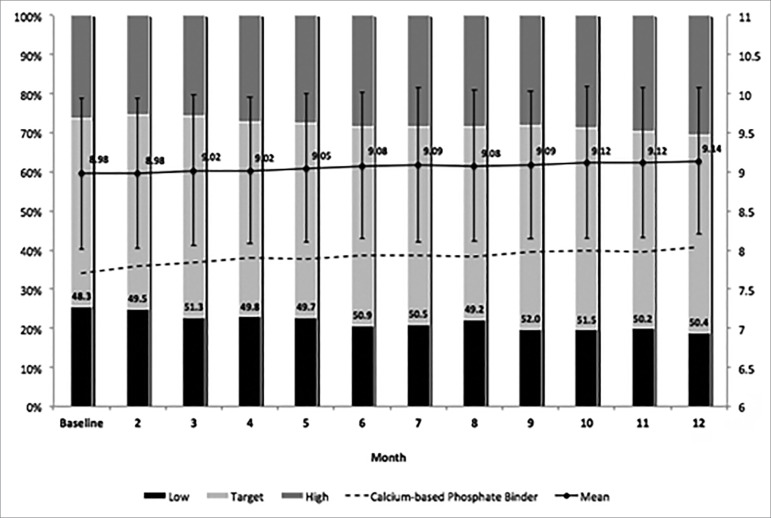
Serum calcium levels (mg/dL) along the first year of dialysis and the use of calcium-based phosphate binders (percentage). Error bars represent standard deviation.

The mean total serum calcium at baseline was 8.98 ± 0.97 mg/dL, it presented a small increase to 9.08 ± 0.93 mg/dL after 6 months, and continued rising to 9.14 ± 0.94 mg/dL after one year of follow-up. At the beginning of the study, 48.3% of our population was within the recommended target for serum calcium level. This prevalence presented a modest increase to 50.9% at 6 months and remained stable thereafter with 50.4% at the first year of therapy. Figure 1 summarizes mean serum calcium levels, the distribution of patients into 3 groups divided according to the KDOQI preferential range, and the prevalence of the use of calcium-based phosphate binders.

### Phosphorus

**Figure 2:**
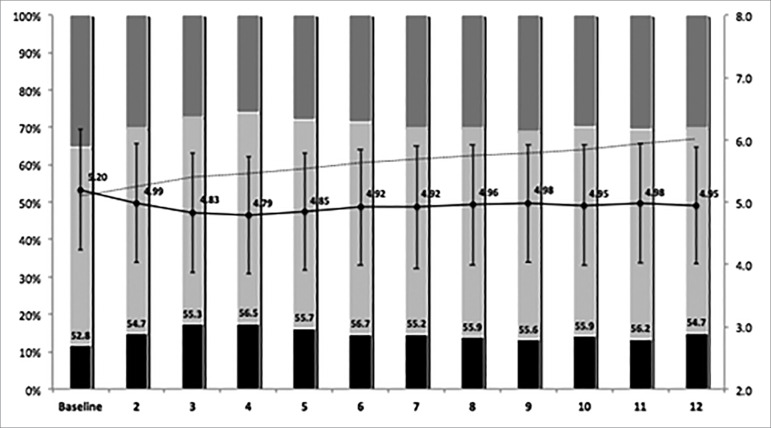
Serum phosphate levels (mg/dL) along the first year of dialysis and the use of any phosphate binder. Error bars represent standard deviation.

The mean serum phosphate at baseline was 5.20 ± 1.65 mg/dL, presenting a small decrease to 4.92 ± 1.55 mg/dL after 6 months, and remaining stable at the end of the first year of follow-up, with 4.95 ± 1.55 mg/dL. At the beginning of the study, 52.8% were within the recommended target for phosphate serum levels, slightly increasing to 56.7% at 6 months, and at the end of the first year on dialysis, 54.7% of the patients were within the target. Figure 2 summarizes mean serum phosphate levels, the distribution of patients into 3 groups divided according to the KDOQI, and the prevalence of the use of any phosphate binder.

### PTH and alkaline phosphatase

**Figure 3:**
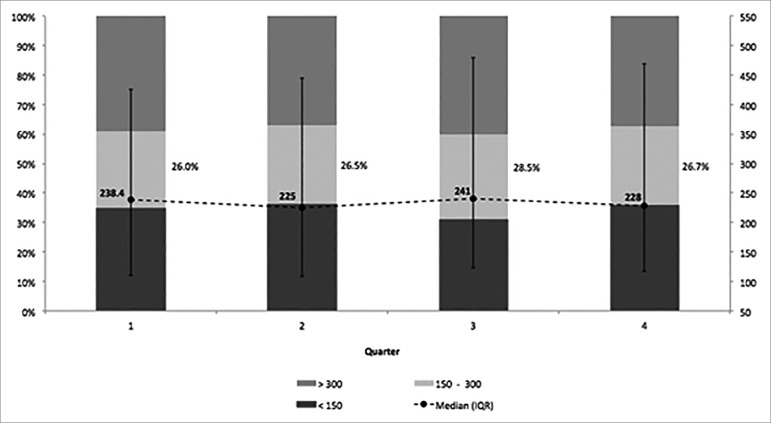
Serum iPTH levels (pg/mL) along the first year of dialysis. Error bars represent interquartile range.

The median iPTH serum level at baseline was 238 (P25% 110 - P75% 426) pg/mL and it remained relatively stable throughout the first year of dialysis, as depicted in Figure 3. The percentage of patients within the KDOQI recommended range was constant, from 26% at the baseline to a maximum of 28.5% in the third quarter after initiation of PD. For ALP, the median at baseline was 98 (IQR 71-154) UI/L and it did not change along the first year of dialysis.

### Prescription of phosphate binders and calcitriol

The prevalence of patients prescribed with calcium-based phosphate binders at baseline was 34.1% and it increased to 40.8% after 1 year. More than 80% of patients were receiving 3.5 meq/L calcium on peritoneal dialysis solution. The prevalence of patients prescribed with any phosphate binder at baseline was 51.7% increasing to 66.9% after 1 year. The registry of the use of calcitriol in the BRAZPD started in 2008 and, at the end of the study, 25% of the patients were taking oral activated vitamin D.

## Discussion

In this large national cohort, we observed the difficulties of PD patients in achieving the CKD-MBD KODQI guideline recommended range^10^. At the end of the first year of therapy, only 50.4, 54.7, and 26.7% of patients were in the suggested range for total calcium, phosphate, and iPTH levels, respectively. Another study from Canada demonstrated the same difficulties in the management of traditional biochemical mineral and bone variables. Only 64.5% of patients had serum phosphate levels within KDOQI targets, 44.5% were within calcium target levels, 28.4% were within PTH suggested range, and 9.4% of PD patients met all 3 targets^16^. We showed in our study that, at the end of the first year on PD, only half of the patients were in the recommended range for serum calcium and phosphorus levels, despite an increase in the prescription of calcium and non-calcium phosphate binders.

Some studies have evaluated the impact of CKD-MBD biochemical abnormalities on mortality in PD patients^8^^,^ 17 ^-^ 19. Avram et al.^17^ observed that lower PTH values were associated with increased mortality, while Rhee et al.^19^, studying 9.244 PD patients in a retrospective cohort study, demonstrated that PTH had a U-shaped association with mortality, with values of 200-700 pg/mL exhibiting the lowest mortality and concentrations < 100 pg/mL, the highest one. Additionally, Liu et al.^8^ demonstrated that the effects of ALP levels may operate as a more consistent predictor of mortality than the traditional calcium, phosphate, and PTH levels, in a large cohort of PD patients in Taiwan. Noordzij et al.^18^, in a prospective cohort study with 586 PD patients, demonstrated that hyperphosphatemia, but not abnormal levels of calcium or iPTH, were associated with increased mortality. Finally, Stevens et al.^20^, in another prospective cohort study with 158 PD patients, observed that only serum phosphate showed significant association with mortality.

Serum calcium and phosphate levels are important biomarkers for the evaluation of CKD-MBD. All guidelines for CKD-MBD recommend special attention to the control of hyper/hypophosphatemia and hyper/hypocalcemia^5^^,^ 10. Disorders of these biomarkers are considered significant risk factors for overall and cardiovascular mortality1 in the dialysis population. During most part of our study, the current guideline was the CKD-MBD KDOQI. Published in 2003 and updated in 2009, this guideline recommended a target for calcium serum between 8.4 and 9.5 mg/dL and phosphate between 3.5 and 5.5 mg/dL. Few studies on dialysis patients showed a small, if any, impact of the KDOQI guideline on the prevalence of calcium and phosphate disorders^17^. We then decided to look at the behavior of these electrolytes along the first year of therapy in a large PD cohort.

Based on the KDOQI guideline, the prevalence of hyperphosphatemia and hypophosphatemia in our cohort at baseline was similar to previous reports from different regions of the world^21^^-^ 23. Importantly, the prevalence of patients on the proposed target for phosphate barely changed along the first year of dialysis, despite an important increase in the proportion of patients taking phosphate binders. Some reasons may have contributed to the difficulty in controlling phosphate serum levels, including a low patient adherence to diet and drug prescription, and a loss of residual renal function. An increase in iPTH with time could also have contributed due to its action on bone resorption. However, iPTH levels remained stable along the first year of dialysis therapy, and probably did not influenced the results.

The prevalence of hyper and hypocalcemia in our cohort was similar to other reports, with a small predominance of hypercalcemia over hypocalcemia 24 ^,^ 25. These disorders have also been associated with increased mortality rates, although less frequently in the setting of PD^25^^,^ 26. The increase of almost 7% in the number of patients with hypercalcemia is likely related to the use of 3.5 mEq/L calcium PD solutions and to the use of calcium-based phosphate binders. Although available to all patients in the country, the 2.5 mEq/L calcium PD solution is not frequently prescribed. Additionally, our group had previously demonstrated that in PD patients with PTH < 150 pg/mL, conversion to low calcium solutions (2.5 mEq/L) appears to be a simple and effective strategy to bring iPTH levels to the range determined by current guidelines^5^ when compared with 3.5 mEq/L calcium PD solutions^27^.

Despite the increase in prevalence of hypercalcemia during the observation period of our study, the percentage of patients taking calcium-based phosphate binders also increased. One possible explanation is related to the bureaucracy involved to get sevelamer hydrochloride from the public health system in some regions of Brazil, where a proof of high serum calcium is required before getting the non-calcium based phosphate binder. Unfortunately, there is no data on this in the BRAZPD database. Changes in the membrane profile may also have contributed to the greater number of patients with hypercalcemia. Exposure to bio-incompatible PD solutions is a factor that may affect the peritoneal membrane and lead to a progressive increase in the transport status. The higher the transport status, the higher the calcium absorbed from the peritoneal cavity^23^.

In our study, only 26.7% of patients had iPTH levels within the range suggested by international guidelines^10^ during the study follow-up. However, the lack of absolute information about the use of calcitriol, nutritional forms of vitamin D, and vitamin D analogs limits the definition of whether further improvement in reaching clinical targets would have been possible.

Our study has some limitations including all those normally related to any observational study such as lack of longitudinal data on residual renal function, lack of data on peritoneal membrane status, no information about the doses and frequency of the phosphate binders prescribed, missing data on iPTH and ALP, and lack of control of patient adherence to medication and diet. Strengths of our study include the large sample size with an excellent external validity, laboratory values of calcium and phosphorus collected monthly, and longitudinal data on the use of phosphate binders.

In conclusion, we observed a high prevalence of biochemical disturbances of CKD-MBD markers in this nation-wide PD cohort. Additionally, initiation of PD was not enough to reduce the high prevalence of calcium and phosphorus disturbances in a public health system that provides free access to dialysis, low-Ca dialysate, calcitriol, and phosphate binders. Further studies are needed to identify the causes behind the difficulties of PD centers in achieving the current recommended targets for serum levels of calcium and phosphorus.
